# Curcumin induces senescence of primary human cells building the vasculature in a DNA damage and ATM-independent manner

**DOI:** 10.1007/s11357-014-9744-y

**Published:** 2015-02-04

**Authors:** Wioleta Grabowska, Karolina Kucharewicz, Maciej Wnuk, Anna Lewinska, Małgorzata Suszek, Dorota Przybylska, Grazyna Mosieniak, Ewa Sikora, Anna Bielak-Zmijewska

**Affiliations:** 1 Department of Biochemistry, Nencki Institute of Experimental Biology, Polish Academy of Sciences, Pasteur Str. 3, 02-093 Warsaw, Poland; 2 Department of Genetics, University of Rzeszow, Kolbuszowa, Poland; 3 Centre of Applied Biotechnology and Basic Sciences, University of Rzeszow, Kolbuszowa, Poland; 4 Department of Biochemistry and Cell Biology, University of Rzeszow, Kolbuszowa, Poland

**Keywords:** Curcumin, Sirtuins, VSMCs, ECs, Aging, Atherosclerosis

## Abstract

Curcumin is considered not only as a supplement of the diet but also as a drug in many types of diseases and even as a potential anti-aging compound. It can reduce inflammation that increases with age and accompanies almost all age-related diseases. It has been suggested that curcumin can play a beneficial role in the cardiovascular system. However, there are also data showing that curcumin can induce senescence in cancer cells, which is a beneficial effect in cancer therapy but an undesirable one in the case of normal cells. It is believed that cellular senescence accompanies age-related changes in the cardiovascular system. The aim of this study was to check if curcumin, in a certain range of concentrations, can induce senescence in cells building the vasculature. We have found that human vascular smooth muscle and endothelial cells derived from aorta are very sensitive to curcumin treatment and can senesce upon treatment with cytostatic doses. We observed characteristic senescence markers but the number of DNA damage foci decreased. Surprisingly, in vascular smooth muscle cell (VSMC) activation of DNA damage response pathway downstream of ataxia-telangiectasia mutated (ATM) was observed. ATM silencing and the supplementation of antioxidants, *N*-acetyl-*L*-cysteine (NAC) or trolox, did not reduce the number of senescent cells. Thus, we have shown that curcumin can induce senescence of cells building the vasculature, which is DNA damage and ATM independent and is not induced by increased reactive oxygen species (ROS) level. We postulate that an increase in the bioavailability of curcumin should be introduced very carefully considering senescence induction as a side effect.

**Electronic supplementary material:**

The online version of this article (doi:10.1007/s11357-014-9744-y) contains supplementary material, which is available to authorized users.

## Introduction

Curcumin has been studied from many years as a potential drug and a protective agent. Abundant evidence from molecular studies as well as from animal models showed efficiency of this factor in treatment of many diseases due to its anti-inflammatory, antioxidative, and anti-cancer activities. Recent studies evaluated curcumin as a potent anti-aging factor and a potent compound in the prevention of age-related diseases, including cardiovascular diseases (CVD) (Shen et al. 2013; Sikora et al. 2010). Aging as well as almost all age-related diseases are accompanied by increased inflammation, which in elderly individuals is usually referred to as persistent low-grade inflammation. It is believed that by lowering the low-grade inflammation, curcumin could potentially delay both organismal aging and age-related diseases, such as neurodegeneration (AD), cancers, or atherosclerosis. Curcumin acts by decreasing the proinflammatory cytokine level and other mediators of inflammation (including NFκB) and by simultaneously increasing the level and activity of proteins involved in antioxidative defense. There are some convincing data showing beneficial effects of curcumin in the vascular system caused by elevation of the level of heme oxygenase-1 (HO-1), NOS, and sirtuins (Yang et al. 2013), enzymes considered as essential for protection of homeostasis in the vascular system.

One of the diseases connected with increased inflammation and classified as an age-related disease is atherosclerosis. The frequency of incidents increases after the age of 50 years. It has been shown that the production of ROS increases as a result of angiotensin II activity and can lead to damage of cells building the vasculature (Wang et al. 2010). It is believed that cellular senescence may be the reason of the organismal aging. This is especially well documented for cells building the vasculature. The senescence of vascular smooth muscle and endothelial cells accompanies both atherosclerosis and hypertension (Gorenne et al. 2006; Minamino et al. 2002; Westhoff et al. 2008). Cells expressing markers of senescence have been found in atherosclerotic plaques. Such cells possess increased number of DNA damage foci containing γH2AX, an increased level of cell cycle inhibitors and higher activity of senescence-associated β-galactosidase (SA-β-gal) (Gorenne et al. 2006). It is documented that curcumin can protect the vascular system and cardiomyocytes from the damaging activity of chemotherapeutic agents, such as doxorubicin (Swamy et al. 2012; Yang et al. 2013). Furthermore, similar to other plant polyphenols, curcumin can lengthen the life span of animal model organisms such as *Caenorhabditis elegans*, *Drosophila melanogaster*, and mouse by modification of the expression of genes involved in cellular senescence. In the case of *C. elegans*, curcumin did not increase the life span of animals with mutated sir2 (mammalian sirtuin 1) (Liao et al. 2011). Long-term studies have shown that curcumin applied in the diet is fully safe and can possess protective activity. Even very high doses of curcumin (8 g/day) did not cause side effects and in the population in which curcumin constitutes a significant part of the diet, the occurrence of some diseases is lower (Cheng et al. 2001). Even though there is no information about the negative effects of curcumin provided in diet, in vitro curcumin can induce apoptosis of both normal and cancer cells (Bielak-Zmijewska et al. 2000). Curcumin belongs to hormetic agents, i.e., its activity depends on the concentration. At low concentration, it has a positive effect for the organism and for particular cells but high concentration may induce detrimental effects, even cell death. Results obtained by us and by others pointed to a new role of curcumin, namely the pro-senescence one. It was observed in cancer HCT116, MCF7, and U2OS cells (Mosieniak et al. 2012) and cancer-associated fibroblasts (CAF) (Hendrayani et al. 2013). It was postulated that in both cases, curcumin could act to protect cells from cancer progression and the effect can be regarded as beneficial and of therapeutic significance. However, it is not difficult to imagine that acceleration of senescence of normal cells could be detrimental. On the other hand, it has also been postulated that senescence of cells building vasculature can protect against atherosclerosis (Muñoz-Espín and Serrano 2014). However, there is still more evidence that senescence of cells building the vasculature supports atherosclerosis.

The pro-senescence activity of curcumin observed in cancer cells inspired us to ask what will happen when primary cells are exposed to cytostatic doses of this compound. Therefore, the aim of our study was to elucidate if curcumin, in concentration which effectively inhibited proliferation without induction of cell death, can induce senescence in primary cells building the vasculature, namely the vascular smooth muscle cells (VSMCs) and endothelial cells (ECs) derived from the human aorta. We showed that cells treated with cytostatic doses of curcumin have undergone senescence in a DNA damage-independent manner. In spite of the lack of DNA damage, activation of the DNA damage response (DDR) pathway was observed in VSMCs. Our results also suggest that transient ROS production, caused by curcumin, does not evoke cellular senescence of these cells and that ataxia-telangiectasia mutated (ATM) does not play a crucial role in curcumin-induced senescence. The mechanism of curcumin-induced senescence needs to be elucidated.

## Materials and methods

### Reagents

Curcumin (C1386) was from Cayman (Ann Arbor, USA); dimethyl sulfoxide (DMSO) (D4540), bovine serum albumin (BSA), 4′,6-diamidino-2-phenylindole (DAPI), and *N*-acetyl-*L*-cysteine (NAC) were purchased from Sigma-Aldrich (Poznan, Poland); DRAQ5 (1,5-bis{[2-(di-methylamino) ethyl] amino}-4, 8-dihydroxyanthracene-9,10-dione) was from BioStatus Limited (Leicestershire, UK); trolox was from Calbiochem (Warsaw, Poland).

### Culture of vascular smooth muscle cells and endothelial cells

Human VMSCs and ECs were purchased from Lonza or from ATCC. VSMCs were cultured in SmBM medium (Lonza, Switzerland) or Vascular Cell Basal Medium (ATTC, LGC, Poland) supplemented as defined by the manufacturer. ECs were cultured in EBM-2 medium (Lonza, Switzerland) or Vascular Cell Basal Medium (ATTC, LGC, Poland) supplemented as defined by the manufacturer. All cells were kept in humidified atmosphere (37 °C and 5 % CO_2_ in the air). The cells were passaged every 3–4 days and were seeded 24 h before treatment at a density of 3–3.5 × 10^3^ cells/cm^2^ (VSMCs) and 10 × 10^3^ cells/cm^2^ (ECs). To eliminate the overlapping effect of replicative senescence in the experiments, only cells in the phase of intensive growth have been used (passage 6–9). Curcumin was dissolved in DMSO and added to the medium to a given final concentration. The DMSO concentration in cell culture did not exceed 0.15 %, which did not influence cell survival. Cell morphology was analyzed in the inverted light microscope.

### Cell proliferation analysis

Cell proliferation was monitored by assessment of cell number in the culture. Cells treated with curcumin were counted in the Neubauer-improved chamber at chosen time points. For DNA synthesis, bromodeoxyuridine assay was used (BrdU, Sigma-Aldrich, Poznan, Poland). BrdU was added to the medium (10 μM) for 24 h and was detected by using primary antibodies against BrdU (Becton Dickinson, Warsaw, Poland). Cells were analyzed under a fluorescence microscope. Cells were counted, and the % of BrdU-positive cells is shown on the graphs.

### Cell cycle and cell granularity analysis

DNA content and cellular granularity analysis were made as described by Korwek et.al. (Korwek et al. 2012). Cells were stained with PI solution, and flow cytometry analysis of 10,000 cells was performed using FACSCalibur Becton Dickinson and the CellQuestPro software.

### Analysis of mitotic cells—MPM2

Cells were treated with curcumin (2.5–15 μM) or nocodazole (100 nM) (Sigma-Aldrich, Poznan, Poland) for the appropriate time, fixed in 4 % PFA and stored in 70 % EtOH at −20 °C. Next, cells were permeabilized and blocked for 30 min in 3 % BSA and 0.1 % Triton X-100 (Sigma-Aldrich, Poznan, Poland) in PBS and stained for 1.5 h with anti-MPM2 antibody (Upstate) diluted 1:1000 in 1 % BSA and 0.1 % Triton X-100 in PBS. Then, cells were washed and stained for 1 h with secondary anti-mouse antibodies conjugated with Alexa Fluor 488 diluted 1:500 in 1 % BSA and 0.1 % Triton X-100 in PBS. Ten thousand cells were analyzed with the use of the flow cytometer (FACSCalibur, Becton Dickinson) and the CellQuestPro software.

### Estimation of senescence-associated β-galactosidase activity

Detection of senescence-associated β-galactosidase (SA-β-gal) activity was performed according to Dimri et al. (Dimri et al. 1995). Cells were analyzed in a light microscope. Then, cells were counted and the % of SA-β-gal-positive cells is shown.

### Measurements of secreted factors

Secretory phenotype was analyzed by ELISA in 1 ml of the culture medium collected from cell culture on 1, 3, and 7 days. Experiments were conducted according to the protocol provided by the manufacturer (R&D Systems, Warsaw, Poland). Levels of cytokines (IL-6, IL-8, VEGF) in the samples were determined with the use of standard curves and normalized to cell number. Absorbance was measured at 450 nm using a Tecan Sunrise spectrophotometer (Tecan) and analyzed with the X-fluor 4 software.

### RayBio human cytokine antibody array

Selected secreted proteins were also analyzed by RayBio Human Cytokine Antibody Array (RayBiotech, Norcross, GA, USA). After 6 days of curcumin treatment, the medium was changed to a fresh one, and 24 h later, it was collected from cell culture. The protein level was normalized according to the cell number present in the dish from which the medium was collected. All required buffers were provided by the producer. Membranes were blocked for 30 min and incubated with conditioned media for 2 h. After washing, biotin-conjugated antibodies were added to each membrane and incubated for 2 h, and then, HRP-conjugated streptavidin was added for the next 2 h. Thereafter, membranes were washed and the signal was developed using West Pico Chemiluminescent Substrate. Membranes were exposed onto medical X-ray film. Description of the selected proteins is in Table 1.

**Table 1:** The positioning of selected proteins analyzed using RayBio Human Cytokine Antibody Array placed at Fig. 2c

	A	B	C	D	E	F	G	H	I	J	K	L	M	N
1	POS	POS	NEG	NEG	IL-1β	IL-1 RI	IL-1 RII	IL-4	IL-6	IL-6 R	IL-8	IL-10	IL-12p40	IL-12p70
2	POS	POS	NEG	NEG	IL-1β	IL-1 RI	IL-1 RII	IL-4	IL-6	IL-6 R	IL-8	IL-10	IL-12p40	IL-12p70
3	IL-13	TACE	Osteopontin	TNF-α	sTNF RI	sTNF RII	GCSF	GRO-α	NAP-2	ENA-78	RANTES	IFN-γ	SDF 1 α	SDF-1 β
4	IL-13	TACE	Osteopontin	TNF-α	sTNF RI	sTNF RII	GCSF	GRO-α	NAP-2	ENA-78	RANTES	IFN-γ	SDF 1 α	SDF-1 β
5	OPG	Fas ligand	EGF	bFGF	VEGF	VEGF-C	VEGF-D	HGF	PDGF-AA	PDGF-AB	TIMP-1	TIMP-2	MMP-2	MMP-3
6	OPG	Fas ligand	EGF	bFGF	VEGF	VEGF-C	VEGF-D	HGF	PDGF-AA	PDGF-AB	TIMP-1	TIMP-2	MMP-2	MMP-3
7	MMP-9	MMP-10	MMP-13	Ferritin	BMP-4	BMP-7	IP-10	IGF-I	IGF-II	IGFBP-4	Leptin	TGF-β2	TGF-β1	TGF-β3
8	MMP-9	MMP-10	MMP-13	Ferritin	BMP-4	BMP-7	IP-10	IGF-I	IGF-II	IGFBP-4	Leptin	TGF-β2	TGF-β1	TGF-β3
9	ICAM-1	ICAM-2	ICAM-3	VCAM-1	Amphiregulin	PAI-I	SCF	SCF R	BLANK	BLANK	BLANK	BLANK	BLANK	POS
10	ICAM-1	ICAM-2	ICAM-3	VCAM-1	Amphiregulin	PAI-I	SCF	SCF R	BLANK	BLANK	BLANK	BLANK	BLANK	POS

### Western blotting analysis

Whole cell protein extracts were prepared according to Laemmli (Laemmli 1970). The primary antibodies used were the following: anti-ATM (1:500), anti-phospho-ATM Ser1981 (1:500), and anti-GAPDH (1:50,000) (Millipore, Warsaw, Poland); anti-p53 (1:500), anti-p21 (1:500), and anti-p16 (1:250) (Santa Cruz Biotechnology, Santa Cruz, USA); anti-phospho-p53 Ser15 (1:250) (Becton Dickinson, Warsaw, Poland); anti-HO-1 (1:1000) (Enzo Life Sciences, Warsaw, Poland); SIRT1 (1:250), phospho-SIRT1 Ser47 (1:250), anti-SIRT2 (1:1000), anti-SIRT3 (1:500), anti-SIRT5 (1:500), anti-SIRT6 (1:1000), anti-SIRT7 (1:250), anti-phospho-p38 Thr180/Tyr182 (1:500), and anti-p38 (1:500) (Cell Signaling Technology, Poznań, Poland); and anti-AGTR1 (1:500) and anti-actin (1:50,000) (Sigma-Aldrich, Poznan, Poland). The respective proteins were detected after incubation with one of the horseradish peroxidase-conjugated secondary antibodies (1:2000) (Dako, Gdynia, Poland), using an ECL system (Thermo Scientific, Warsaw, Poland), according to the manufacturer’s instructions.

### Global DNA methylation assay

DNA methylation was estimated as the 5-methyl-2′-deoxycytidine (5-mdC) level using high-performance liquid chromatography (HPLC) as described elsewhere (Potocki et al. 2012). For global DNA methylation inhibition control, a 24-h cell treatment with 5 μM 5-aza-2′-deoxycytidine (5-aza-dC) was used.

### Immunocytochemistry

For detection of 53BP1 foci, cells were fixed with 70 % ethanol and next incubated with primary anti-53BP1 antibodies (Novus Biological, Cambridge UK) diluted 1:500 and with Alexa 488 secondary antibody (Life Technology, Eugene, USA), 1:500. DNA was stained with DAPI. 53BP1 foci were visualized under a fluorescence microscope.

### Measurement of ROS level

The steady-state level of reactive oxygen species (ROS) in the cell culture medium and intracellular superoxide production both total and mitochondrial were measured with 2′,7′-dichlorodihydrofluorescein diacetate (H_2_DCF-DA), dihydroethidium, and MitoSOX™, respectively, as described previously (Mytych et al. 2014).

### Mitochondrial membrane potential

The mitochondrial membrane potential (MMP) was measured with rhodamine G6 as described previously (Mytych et al. 2014).

### Cytokinesis-block micronucleus assay

The evaluation of micronuclei generation was performed using a BD™ Gentest Micronucleus Assay Kit (Becton Dickinson, Warsaw, Poland) following the standard protocol provided by the manufacturer as described previously (Bielak-Zmijewska et al. 2014).

### Silencing of the *ATM* gene

To downregulate *ATM* expression, the cells were transfected with 30 nM siRNA (*ATM* or negative) (Life Technologies, Warsaw, Poland) using Lipofectamine 2000 (Life Technologies, Warsaw, Poland). Transfection was performed according to the manufacturer’s protocol. About 24 h after transfection, medium was replaced with fresh one and cells were cultured up to 7 days in the presence of curcumin.

### Statistical analysis

Statistical analysis was performed using two-tailed Student *t* test or ANOVA with post hoc testing using a Dunnett’s multiple comparison test. Data are presented as a mean ± SD. A value of *p* < 0.05 was considered statistically significant (*p < 0.05, **p < 0.01, ***p < 0.001). All graphs show the mean results from at least three independent experiments.

## Results

### VSMC and EC sensitivity to curcumin treatment

**Fig. 1:**
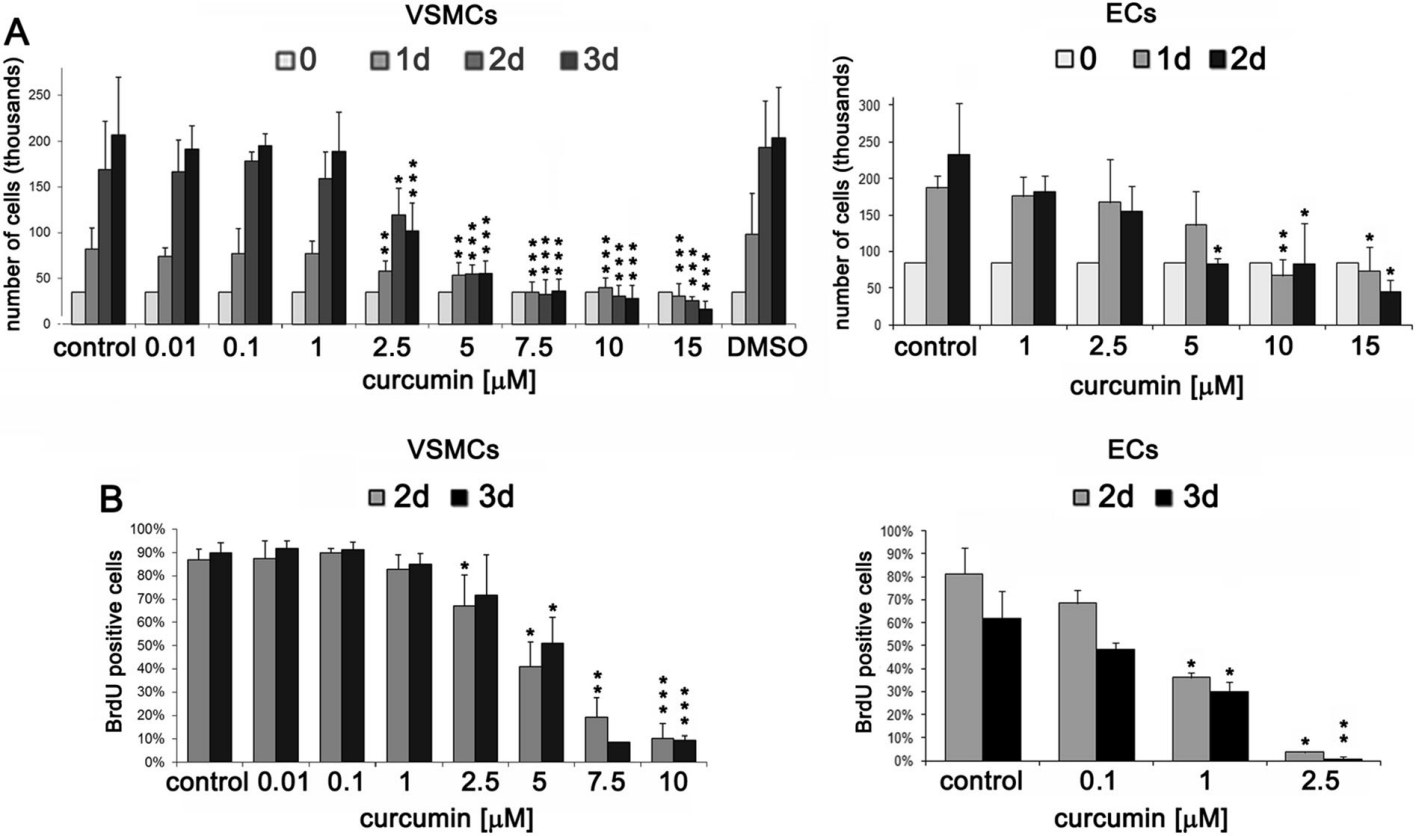
Proliferation potential of VSMCs and ECs treated with different concentrations of curcumin. **a** Proliferation potential measured by counting VSMCs and ECs (curcumin between 10 nM and 15 μM). The concentration of DMSO in control (0.15 %) corresponded to the concentration of DMSO added to the culture medium with the highest curcumin dose. **b** Estimation of proliferation rate by measurement of DNA synthesis as BrdU incorporation in VSMCs and ECs (curcumin between 10 nM and 10 μM). The percentage of BrdU-positive cells is presented on the graphs. *1-3d* 1–3 days after curcumin treatment. *Error bars* indicate SD, *n* = 3 or more. *t* test, *p < 0.05, **p < 0.01, ***p < 0.001, compared to control cells.

VSMCs and ECs derived from the human aorta have been studied for their sensitivity to curcumin (Fig. 1a). Already at 10 μM concentration of curcumin, VSMCs started to die. Cells stopped to proliferate in the presence of 5 μM curcumin. Analysis of BrdU incorporation revealed that the number of cells that were able to synthesize DNA within 18 h decreased at 2.5 μM curcumin, and at 7.5 μM, the inhibition seemed to be more effective (Fig. 1b). For ECs, the 2.5 μM concentration inhibited cell proliferation (the incorporation of BrdU was almost totally inhibited) (Fig. 1a, b) while 10 μM and higher concentrations were cytotoxic. Our results revealed that concentrations below 1 μM did not influence VSMC proliferation but slightly inhibited that of ECs, as shown by cell counting and BrdU incorporation (Fig. 1b). In spite of the cell death observed after curcumin treatment, the sub-G1 fraction of cells corresponding to the population of apoptotic cells with fragmented DNA (level lower than 2C) was insignificant for both VSMCs (about 2 %) and ECs (about 8 %) (not shown).

For VSMCs and ECs, the cell cycle analysis was performed (Online Resource 1). In VSMCs, the range of curcumin concentrations (between 5 and 15 μM) induced changes in the cell cycle, manifested by increased population of cells in the G2/M phase. In ECs, no changes in the proportion of cells in the particular phases of the cell cycle were observed.

In order to check whether cells containing 4C DNA accumulated in the G2 or M phase of the cell cycle, we analyzed the percentage of MPM2-positive cells (Online Resource 2). Cells treated with nocodazole, the inhibitor of microtubule polymerization which arrests cells during mitosis, served as a positive control. For untreated cells, the percentage of mitotic cells was about 2–3 %. We showed that curcumin did not increase the number of cells in mitosis. The observed low percentage of MPM2-positive cells suggests that curcumin arrested cells in the G2 phase of the cell cycle.

### Curcumin induces cellular senescence of human VSMCs and ECs in vitro

**Fig. 2:**
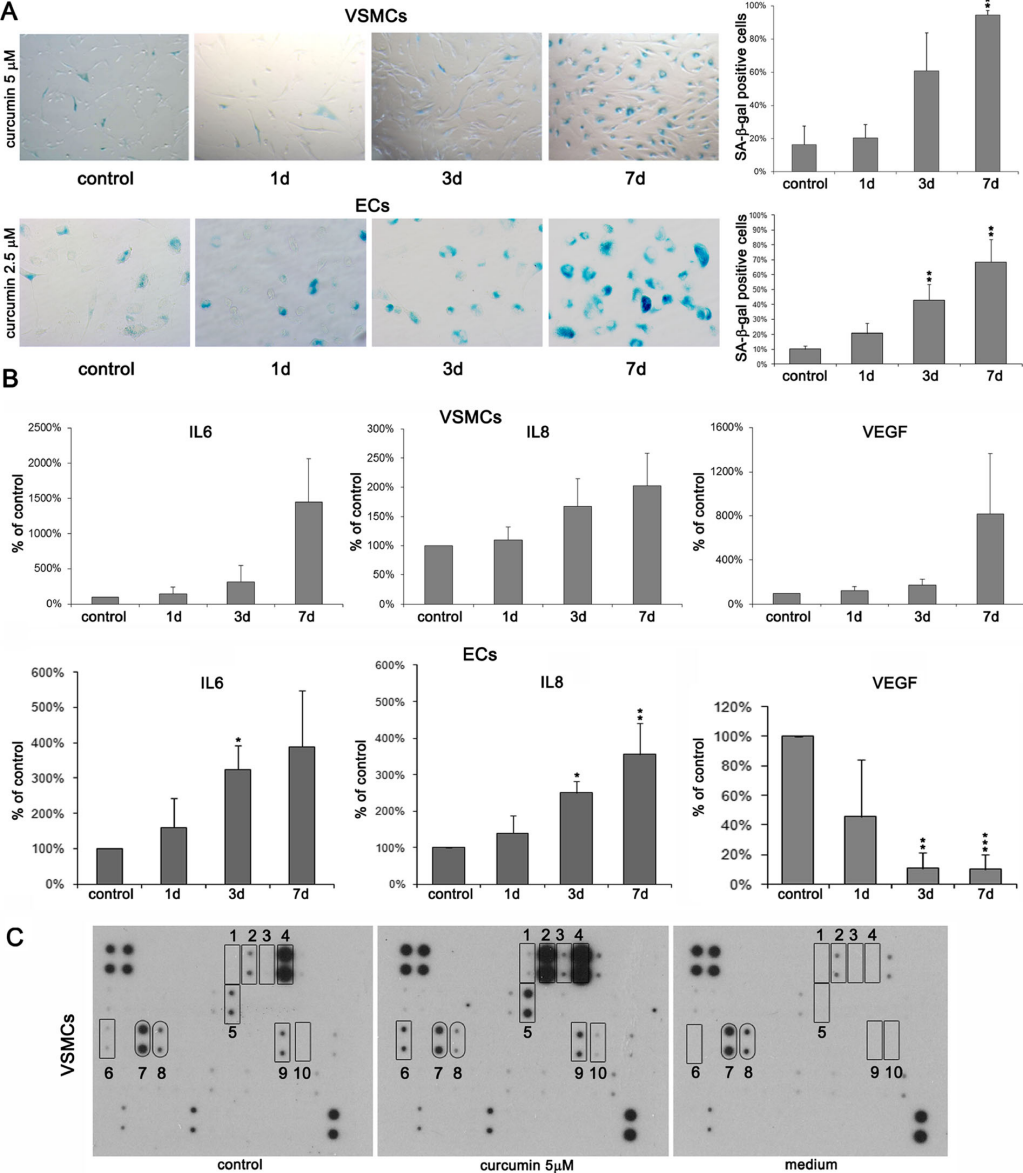
Analysis of the selected markers of senescence upon treatment with cytostatic doses of curcumin (5 μM for VSMCs and 2.5 μM for ECs). **a** SA-β-gal activity in VSMCs and ECs. The graphs with the percentage of SA-β-gal-positive cells and representative pictures are shown. Magnification ×200. **b** Secretory phenotype (*SASP*) of VSMCs and ECs. The level of IL-6, IL-8, and VEGF was shown. *1-7d* 1–7 days after curcumin treatment. *Error bars* indicate SD, *n* = 3 or more. *t* test, *p < 0.05, **p < 0.01, ***p < 0.001, compared to control cells. **c** Changes in selected secreted protein level in VSMCs measured using RayBio Human Cytokine Antibody Array. The respective membranes represent control cells, cells treated with 5 μM curcumin, and pure medium to exclude unspecific changes in protein level: *1* IL-4, *2* IL-6, *3* IL-GR, *4* IL-8, *5* GROα, *6* OPG, *7* EGF, *8* bFGF, *9* TIMP1, and *10* TIMP2. The level of the secreted proteins was estimated in culture medium collected after 24 h after culturing from control and curcumin-treated cells (6 days with curcumin and 24 h in a fresh medium). Additional control was performed with medium that was not used for cell culture. The level of proteins which increased is marked with *rectangles* and those which level decreased with *rounded rectangles*.

To study the long-term effect of cytostatic concentrations of curcumin, VSMCs and ECs were cultured in the presence of curcumin for a few days. For VSMCs 5–7.5 μM and for ECs 2.5–5 μM, concentrations have been chosen. Cells treated with these concentrations of curcumin demonstrated some markers characteristic for senescence. In both types of cells, increased SA-β-gal activity was observed (Fig. 2a). After 7 days, about 80 % of VSMCs (Fig. 2a) and about 70 % of ECs (Fig. 2a) were SA-β-gal positive. The secretory phenotype was manifested by increased production of IL-6 and IL-8 in both types of cells (Fig. 2b). In the case of VEGF, increased secretion was observed only in VSMCs, while the level of this growth factor decreased in ECs. In VSMCs, the level of selected secreted proteins after curcumin treatment was studied using the RayBio Human Cytokine Antibody Array (Fig. 2c and Table 1). We found that the level of eight proteins increased in curcumin-senescent cells and the level of two proteins decreased. Elevated proteins were IL-4 (1), IL-6 (2), IL-GR (3), IL-8 (4), growth-related oncogene α (GROα) (5), osteoprotegerin (OPG) (6), tissue inhibitors of metalloproteinases 1 (TIMP1) (9), TIMP2 (10), and those alleviated were EGF (7) and bFGF (8). The inhibition of proliferation was irreversible. After removal of curcumin, cells were cultured for 3–4 weeks without resuming proliferation. Therefore, we concluded that curcumin induced senescence.

**Fig. 3:**
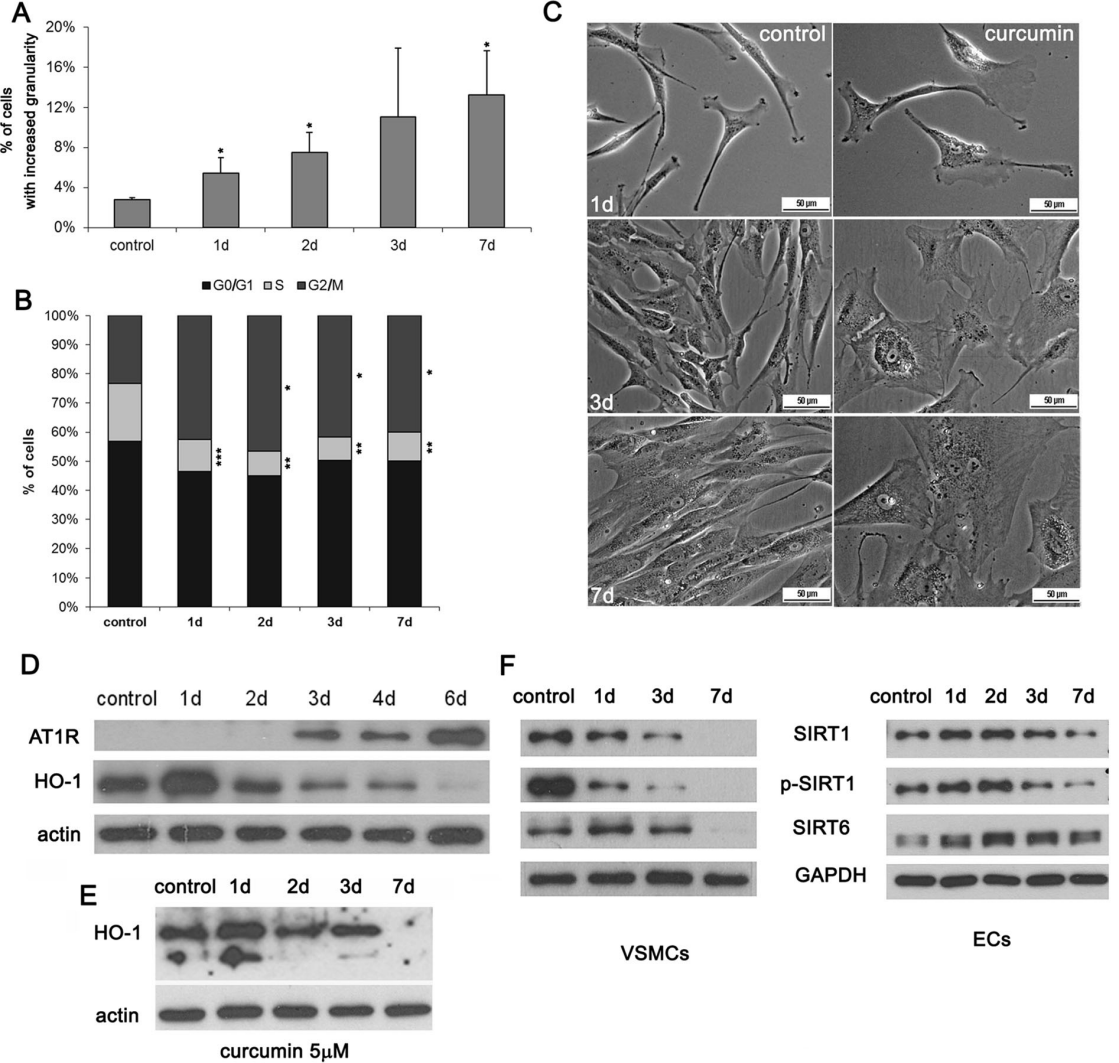
Analysis of selected senescence markers upon cytostatic curcumin doses. **a** Granularity of VSMCs upon 7.5 μM curcumin expressed as the percentage of cells with increased granularity. **b** Cell cycle analysis of VSMCs treated with 7.5 μM curcumin. The graphs show % of cells in the particular phases of the cell cycle. *1-7d* 1–7 days after curcumin treatment. *Error bars* indicate SD, *n* = 3 or more. *t* test, *p < 0.05, **p < 0.01, ***p < 0.001, compared to control cells. **c** Morphology of VSMCs treated with 5 μM curcumin. Representative pictures are shown. **d** Western blot analysis of AT1R and HO-1 during curcumin-induced senescence of VSMCs (5 μM curcumin). Actin served as a loading control. **e** Western blot analysis of HO-1 during curcumin-induced senescence of ECs (2.5 μM curcumin). Actin served as a loading control. **f** Western blot analysis of sirtuin 1 and 6 levels and phosphorylation of sirtuin 1 in VSMCs (5 μM curcumin) and ECs (2.5 μM curcumin).

During curcumin-induced senescence of VSMCs, enhanced cellular granularity was observed (Fig. 3a). It was detected in about 12 % of cells after 7-day treatment. No spectacular changes were observed in ECs. Cell accumulation in the G2 phase of the cell cycle accompanied VSMC senescence (Fig. 3b). We observed also an increased cell size and changed morphology of the cells (Fig. 3c). In VSMCs, the level of AT1R, the receptor of angiotensin II, increased after curcumin treatment (Fig. 3d). Opposite situation was observed for HO-1, an enzyme responsible for antioxidative defense, especially important for EC homeostasis. The level of this enzyme decreased in both types of cells upon curcumin treatment (Fig 3d, e). We did not observe any changes in global DNA methylation (Online Resource 3) in VSMCs as a result of curcumin-induced senescence. We also analyzed the level of two sirtuins involved, among others, in DNA repair, longevity, and proper functioning of the vascular system, namely sirtuin 1 (total and phosphorylated form) and sirtuin 6 (Fig. 3f). In VSMCs, the level of both sirtuins decreased and was undetectable after 7 days. In ECs, a transient increase was observed, but after 7 days, the level of sirtuin 1 decreased visibly while that of sirtuin 6 only slightly and was still higher than the starting point level in untreated cells.

### Mechanism of cell senescence induced by curcumin treatment

#### Role of DNA damage in curcumin-induced senescence

**Fig. 4:**
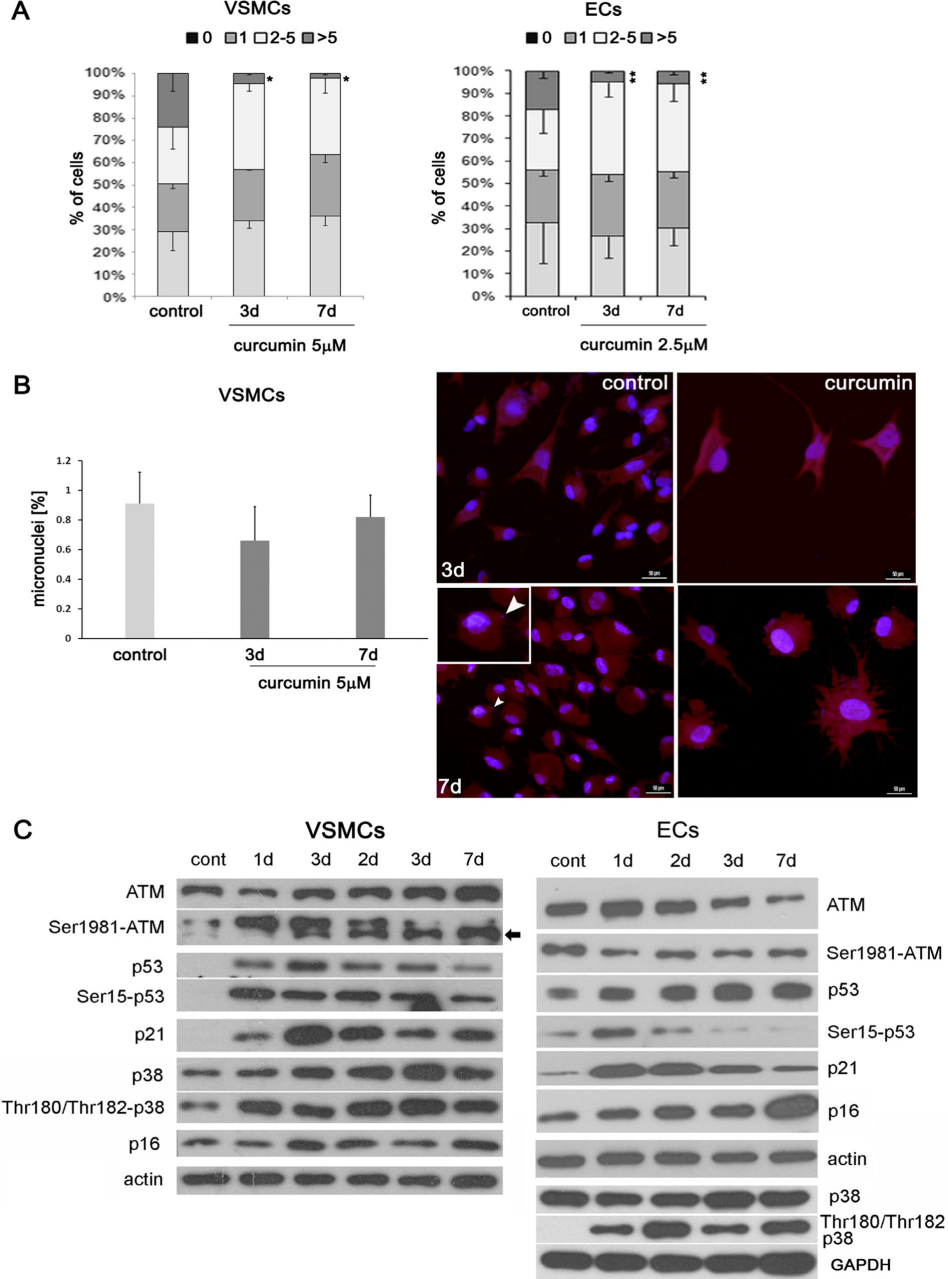
The role of DNA damage in curcumin-induced senescence of VSMCs and ECs. **a** DNA damage expressed as the number of DNA DSBs visualized by 53BP1 immunocytochemistry in VSMCs and ECs (curcumin 5 and 2.5 μM, respectively). *0* cells without DNA damage, *1* with only one focus, *2-5* with number of foci between 2 and 5, *>5* cells with more than five foci. *1-7d* 1–7 days after curcumin treatment. *Error bars* indicate SD, *n* = 3 or more. *t* test, *p < 0.05, **p < 0.01, ***p < 0.001, compared to control cells. **b** Micronuclei generation in VSMCs upon curcumin treatment. Graph and representative pictures are shown. *Arrowhead* indicates a cell with a micronucleus. **c** Western blot analysis of proteins belonging to DDR pathway and proteins involved in cellular senescence.

DNA damage is one of the most common causes of the stress-induced premature senescence. We analyzed the number of DNA damage foci by immunocytochemical detection of the 53BP1 protein (Fig. 4a). We distinguished four categories of cells with damaged DNA, depending on the number of foci (one focus represents one double-strand break). The first category included cells without DNA double-strand break (DSB); the second, cells with only one focus; the third, cells with the number of foci between 2 and 5; and the fourth, cells with more than five foci. After curcumin treatment in the case of both VSMCs and ECs, the number of cells with more than five foci was lower than in control cells. Besides, in the case of VSMCs, the number of cells without DNA DSB increased. Moreover, we showed that, unlike genotoxic agents, curcumin did not induce micronucleation (Fig. 4b). However, curcumin induced the DNA damage response pathway in VSMCs (Fig. 4c). For example, the elevated level of ATM was observed and it correlated with an increase in the level of the phosphorylated form of this protein. Also, the level of total and phosphorylated p53 increased, as well as the level of its target protein, p21, an inhibitor of the cyclin-dependent kinases. No changes were observed in the level of another cell cycle inhibitor, namely p16, but the elevation of the level and phosphorylation of p38, a kinase belonging to MAPK and involved in the senescence growth arrest, accompanied the curcumin-induced senescence. In the case of ECs (Fig. 4c), ATM level was relatively high in control cells and decreased upon curcumin treatment, but the level of the phosphorylated form was constant. The level of p53 increased but its phosphorylated form was observed only after 1-day treatment then gradually decreased. This was consistent with the transient elevation of p21. The p16 level increased gradually, and the level of phosphorylation of p38 increased after 24 h but the level of p38 did not change.

As it has been shown above, curcumin induces DNA damage-independent activation of the DDR pathway in VSMCs. However, in ECs, DDR pathway activation is not observed, but in both types of cells, senescence is DNA damage independent.

#### Role of ROS in curcumin-induced senescence of VSMCs

Because we showed that DNA damage was not the cause of the senescence, we asked what could induce the DDR pathway in VSMCs and, in consequence, be responsible for senescence induction. There are data suggesting that ATM can be activated directly by oxidative stress (Guo et al. 2010).

**Fig. 5:**
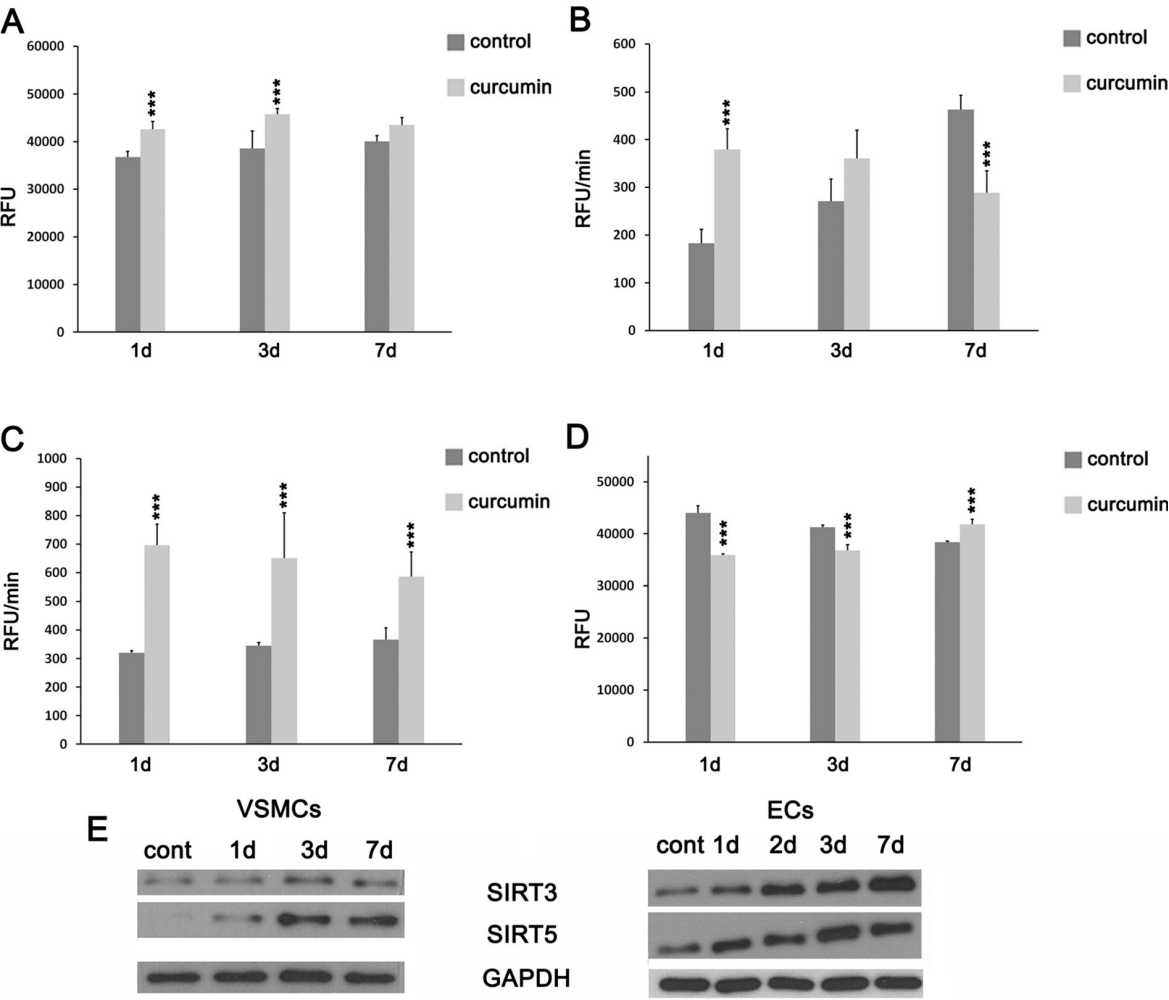
Oxidative stress parameters of VSMCs treated with curcumin. **a** Total ROS level in the culture medium (5 μM curcumin). Data are presented as relative fluorescence unit (*RFU*). **b** Intracellular total superoxide production (5 μM curcumin). RFU per minute. **c** Intracellular mitochondrial superoxide production (5 μM curcumin). RFU per minute. **d** Changes in mitochondrial membrane potential (*MMP*) (5 μM curcumin). Data are presented as RFU. *1-7d* 1–7 days after curcumin treatment. *Error bars* indicate SD, *n* = 3 or more. ANOVA with post hoc testing using a Dunnett’s test, *p < 0.05, **p < 0.01, ***p < 0.001, compared to control cells. **e** Western blot analysis of mitochondrial sirtuin 3 and 5 (7.5 μM curcumin for VSMCs and 2.5 μM for ECs).

The first step to study the mechanism of senescence induction by curcumin in VSMCs was to measure the level of ROS production. We observed an increased steady-state level of total ROS in the culture medium of curcumin-treated cells (Fig. 5a). Intracellular superoxide production in untreated cells increased during the culture, but in curcumin-treated cells, the production was elevated only after 1 and 3 days in comparison to the control cells (Fig. 5b). Seven days after treatment, it was lower than in the control one. An increase in the intracellular mitochondrial superoxide production was observed during the whole time of treatment in comparison to control cells, where the production was constant during the culture period (Fig. 5c). Curcumin mediated also a change in the mitochondrial membrane potential (Fig. 5d). The mitochondrial membrane potential gradually decreased in untreated cells. In curcumin-treated cells, the mitochondrial membrane potential on the first and the third days was lower than in the control cells but on the seventh day was higher than in the control. We analyzed the level of sirtuins present in mitochondria, which are involved in energy homeostasis, mitochondrial biogenesis, and reduction of ROS and participate in cardiac homeostasis as well as aging (Park et al. 2013). In both types of cells, the elevation of the level of sirtuin 3 and 5 was observed (Fig. 5e).

**Fig. 6:**
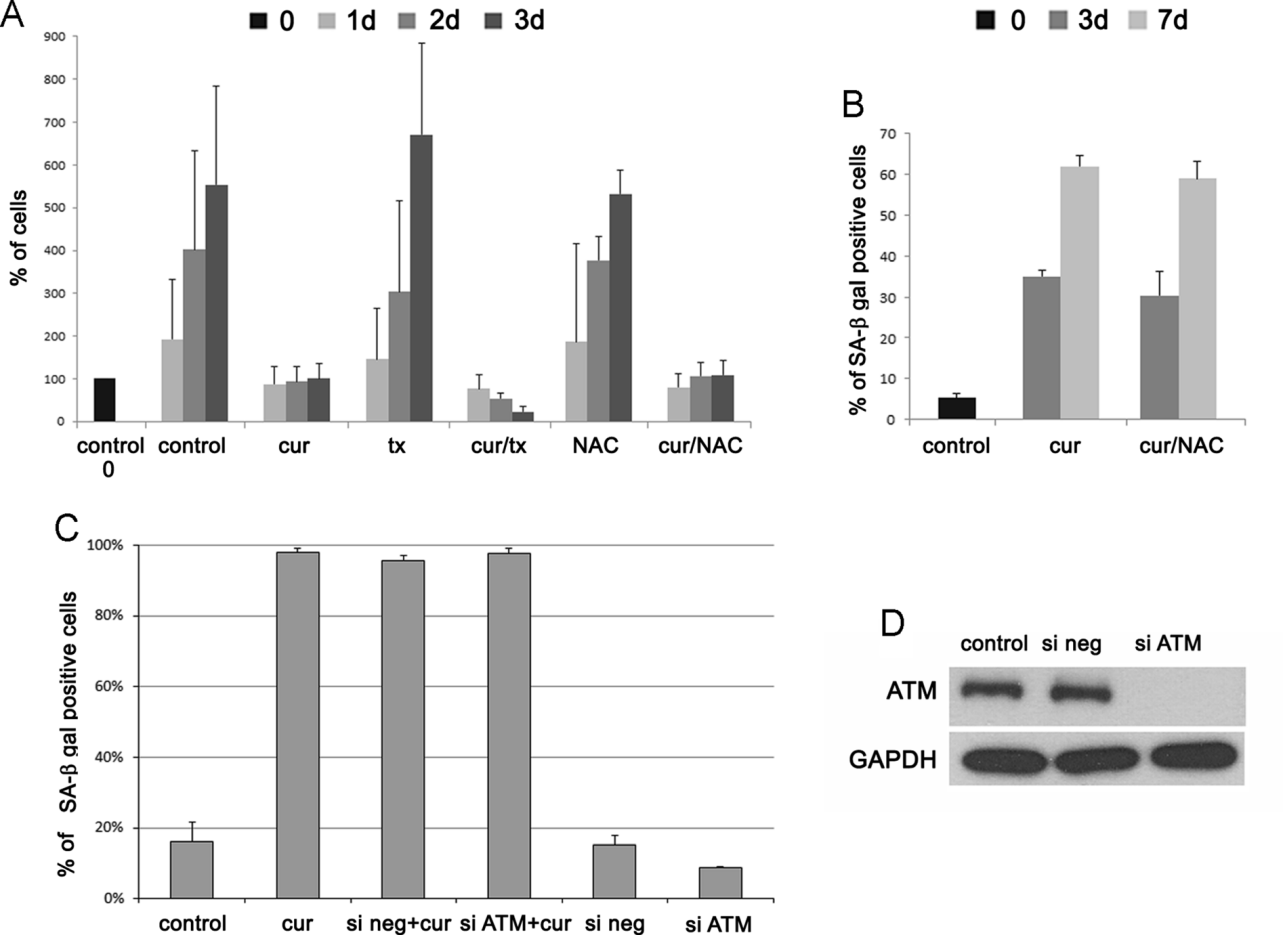
The role of ROS and ATM in curcumin-induced senescence of VSMCs. **a** The proliferation rate of VSMCs pretreated for 30 min with antioxidants NAC (1 μM) and trolox (*tx*) (500 nM) and next treated with 7.5 μM curcumin (cell counting). The number of untreated cells is taken as 100 %. The concentrations of antioxidants were chosen experimentally and doses which did not affect proliferation were used. **b** The percentage of SA-β-gal-positive cells after 30 min pretreatment with NAC (1 μM) and subsequent treatment with 7.5 μM curcumin. **c** Influence of ATM silencing on the ability of VSMCs to undergo senescence. The number of SA-β-gal-positive cells as a result of curcumin treatment and in cells which were transfected with siRNA (negative or ATM) without treatment with curcumin is presented on the graph. *1-7d* 1–7 days after curcumin treatment. *Error bars* indicate SD, n = 3 or more. **d** The efficiency of ATM silencing (after 48 h) is shown on the Western blot.

As we found out, in VSMCs, curcumin increased the intracellular ROS production even on the third day of treatment. Increased ROS production resulted probably from the increased mitochondrial superoxide production. It was inversely correlated with mitochondrial membrane potential. To establish the role of ROS in curcumin-induced senescence of VSMCs, we supplemented the culture medium of cells treated with curcumin with classic antioxidants, namely trolox and NAC (*N*-Acetyl-*L*-cysteine) (30-min pretreatment). Such concentrations of antioxidants were chosen experimentally so as not to impair cell proliferation. Our results revealed that neither trolox nor NAC improved proliferation of cells treated with curcumin nor reduced the number of senescent cells (Fig. 6a, b). We did not observe any differences between cells which were treated with NAC together with curcumin and cells which were only treated with curcumin. Trolox together with curcumin caused an elevated rate of cell death. We analyzed the DDR pathway as well as the proteins markers of senescence established earlier for this type of cells, namely AT1R and HO-1. We could not observe any differences in the protein levels between cells which were only treated with curcumin and those which were pretreated with NAC or trolox (not show).

Our results showed that curcumin-induced senescence of VSMCs was accompanied by oxidative stress, but the antioxidant intervention failed to overcome the pro-senescent activity of curcumin.

#### Role of ATM in curcumin-induced senescence

We also checked if ATM was in general responsible for curcumin-induced senescence of VSMCs. To this end, we silenced the ATM protein with specific siRNA and we analyzed the number of senescent cells upon curcumin treatment. The silencing of ATM was effective but did not reduce the number of senescent cells, as it has been shown by SA-β-gal analysis after 7-day treatment (Fig. 6c, d). Cells transfected with siRNA (ATM or scramble) and not treated with curcumin proliferated in a similar manner as control cells, and the number of SA-β-gal-positive cells was almost the same. It suggested that curcumin-induced cell senescence is ATM independent.

## Discussion

The results of this study revealed that curcumin’s effect on VSMCs and ECs is concentration and cell type dependent. The cytostatic concentrations for ECs were between 2.5 and 5 μM, and for VSMCs between 5 and 7.5 μM. Higher concentrations were cytotoxic. Interestingly, 10 μM concentration was not cytotoxic for fibroblasts and human HaCaT keratinocytes (Hendrayani et al. 2013; Zhao et al. 2013).

We analyzed the impact of cytostatic concentrations till the seventh day of culture. Such concentrations of curcumin induced cellular senescence in both types of cells. Curcumin-induced senescence was evidenced by altered cell morphology, increased granularity, and accumulation of VSMCs in the G2 phase of the cell cycle. This has been already described as one of the markers of stress-induced premature senescence (SIPS) in VSMCs (Bielak-Zmijewska et al. 2014). MPM2 analysis revealed only few mitotic cells after curcumin treatment and suggested that cells were accumulated in the G2 phase of the cell cycle, not in mitosis. It has been observed in cells undergoing prolonged mitosis that the proteins protecting telomeres were dispersed which provoked senescence induction (Hayashi et al. 2012). We have shown earlier that curcumin caused a mitotic spindle dysfunction by its impact on microtubules (Bielak-Zmijewska et al. 2010), and this could be the reason of a prolonged mitotic arrest and telomere proteins diffusion. However, on the basis of the current results, we can exclude that curcumin induced senescence in VSMCs and ECs by mitosis prolongation.

As a result of curcumin-induced senescence, an increased level of angiotensin II receptor, AT1R, was observed (only in VSMCs). AT1R is a cell-type specific marker of VSMC senescence (Stegbauer and Coffman 2011). In both ECs and VSMCs, the level of HO-1 decreased, which pointed to the impaired antioxidative defense. It has been observed earlier in carotid bodies in rats that the level of HO-1 decreased with age (Di Giulio et al. 2009). Moreover, increased HO-1 level protected cells from senescence (Clérigues et al. 2012). It was documented that curcumin induced HO-1 in a short-term cell treatment (Lima et al. 2011), and this effect is considered beneficial in both cardiovascular protection and anti-aging intervention.

We observed increased activity of SA-β-gal and senescence-associated secretory phenotype (SASP) manifested by an increased level of IL-6 and IL-8. It has been shown earlier that curcumin reduces inflammation by decreasing IL-6 (Cho et al. 2007) and IL-8 levels (Cohen et al. 2009). Moreover, in cancer-associated fibroblasts (CAF) undergoing senescence as a result of curcumin treatment, SASP has not been observed (Hendrayani et al. 2013). In VSMCs, also, the production of VEGF increased, but in ECs, an opposite tendency was observed. We speculate that the reduction of VEGF in ECs is a cell-type-specific marker of senescence, and the elevation in VSMCs is the result of SASP (increased VEGF is a feature of SASP). It has been described that decreased level of VEGF is a characteristic feature of EC senescence (Ahluwalia et al. 2014). There are also data showing that curcumin was able to reduce the level of this growth factor and in this manner protected against angiogenesis-accompanied metastasis (Gururaj et al. 2002; Shao et al. 2002). We also analyzed the changes in the level of selected secreted proteins during curcumin-induced senescence in VSMCs. The level of some of them increased, for example those associated with inflammation: IL-4 and GROα or inhibitors of metalloproteinases, TIMP1 and TIMP2.

Interesting is the result showing the increased secretion of osteoprotegerin (OPG) by curcumin. It has been evidenced that this protein protects cells from calcification, which is one of the characteristic features of VSMC senescence (Bennett et al. 2006). Calcification is strongly correlated with atherosclerosis, and the protective effect of curcumin against vascular cells mineralization could be considered as a new beneficial activity of this compound. Our preliminary results suggest that the expression of OPG was maintained and was even higher in cells undergoing curcumin-induced senescence.

Since cells senesced after curcumin treatment, we investigated whether free radicals play a role in this process. It is well documented that curcumin possesses antioxidative activity but there are also data suggesting that it can induce a prooxidative response (Sandur et al. 2007). Our data have shown that curcumin increased ROS level in the medium, mitochondrial superoxide production, and intracellular ROS level but only during the first days of treatment. Subsequently, the level of cellular ROS was significantly lower than in control cells. However, mitochondrial superoxide production was still higher compared to untreated cells, which together with decreased HO-1 expression may indicate that curcumin induced oxidative stress and impaired the antioxidative defense during cellular senescence. Opposite correlation was observed for the mitochondrial membrane potential, which decreased during the first 3 days and increased at day 7 which might be interpreted as adaptation to a long-term exposure to cellular (oxidative) stress. The increased mitochondrial membrane potential in senescence was observed earlier (Sugrue et al. 1999). Interestingly, curcumin increased the level of mitochondrial sirtuins 3 and 5. These proteins are activated in response to stress conditions, and sirtuin 3 appears to be an antioxidant protein, because it can reduce ROS by deacetylation which leads to the activation of superoxide dismutase 2 (SOD2) (Chen et al. 2011). Sirtuin 3 is involved in aging and cardiac homeostasis, and its overexpression is protective against cardiac hypertrophy while depletion enhances the susceptibility to hypertrophy (Sundaresan et al. 2009). We cannot exclude that the elevation of mitochondrial sirtuins is characteristic for dual curcumin action. It could represent the protective mechanism induced by an increased ROS production as a result of curcumin treatment (curcumin simultaneously leads to ROS generation and activates some proteins which participate in an antioxidative defense). It has been shown by others that the level of sirtuin 3 decreased with age (Brown et al. 2013). Moreover, it has been shown that ectopically expressed sirtuin 3 partially reverses the age-associated functional decline of murine hematopoietic stem cells (Brown et al. 2013). The role of mitochondrial sirtuins in senescence needs to be elucidated.

To establish the mechanism of senescence induction upon curcumin treatment, we analyzed the ability of curcumin to induce DNA damage. DNA damage is one of the most common causes of cellular senescence (Sedelnikova et al. 2004) what was also shown by us in VSMCs treated with doxorubicin (Bielak-Zmijewska et al. 2014). There are contradictory data concerning curcumin; in some papers, evidence can be found that curcumin induces DNA damage (Blasiak et al. 1999; Cao et al. 2006) but our earlier results (Bielak-Zmijewska et al. 2000) as well the result obtained by others (Hendrayani et al. 2013) show that it does not. Surprisingly, we found out that cells senescing upon curcumin treatment possessed less DNA damage foci than untreated cells. However, the DNA damage response (DDR) pathway, namely ATM, and p53 were activated in VSMCs. Cellular senescence with pseudo-DNA damage response has already been described, and the authors suggested that activation of the DDR pathway was rather a consequence of senescence and not the cause. (Pospelova et al. 2009).

We studied also the level of other proteins involved in cellular senescence, such as the cell cycle inhibitor p16. The level of p16 increased in ECs but was unchanged in VSMCs. The p38 protein is activated normally as a result of acute cellular stress and leads to cell growth arrest due to its ability to activate both p53 and pRb/p16 (Freund et al. 2011). It has been shown that p38 activity is necessary and sufficient for development of SASP in DNA damage-induced senescence, independently of the DDR pathway. In our experiments, despite the lack of DNA DSBs, the level of phosphorylated p38 increased in both types of cells. It has been shown that curcumin was able to inhibit p38 protein expression (Shehzad et al. 2010).

There are data showing that the DDR pathway activation can be the result of direct activation of ATM by ROS (Guo et al. 2010), and we established that curcumin induced ROS production. However, our results showed that neither trolox nor NAC reduced the percentage of senescent cells and influenced the level of proteins from the DDR pathway. Moreover, cells treated with curcumin together with trolox showed increased mortality. Such effect was earlier shown for cancer cells where trolox enhanced the cytotoxicity of curcumin (Zheng et al. 2012). We also silenced ATM, and it did not prevent VSMC senescence. This suggests that curcumin-induced senescence is ATM independent. ATM-independent senescence was observed by us also in ECs in which no increased level or phosphorylation of ATM was detected. ATM-independent senescence has been shown in primary fibroblasts derived from patients with ataxia-telangiectasia and was induced by p38 activation. (Barascu et al. 2012).

We analyzed the impact of curcumin on sirtuins level. Sirtuins are NAD-dependent deacetylases. These enzymes regulate transcription of genes and are involved in many cellular processes including inflammation, DNA repair, telomere structure and genome integrity maintenance and are considered as anti-aging agents (Morris 2013). It is documented that the level of sirtuin 1 and 6 as well as the efficiency of both DNA repair systems, homologous recombination (HR) and non-homologous end joining (NHEJ) decreased during aging (Mao et al. 2012; Seluanov et al. 2004). On the other hand, it is believed that curcumin increases the level of some sirtuins and in this manner exerts its beneficial effect for the organism. In cells senescing upon curcumin treatment, the level of sirtuin 1 and 6 decreased.

This discrepancy could be due to the overlapping of two effects, i.e., one characteristic for curcumin activity (early response) and second, resulting from senescence (late response). Such conclusion seems justified since most often, the initial response was typical for the known beneficial activity of curcumin (increased level of sirtuins 1 and 6 or HO-1 at day 1) and only later on became characteristic for senescence.

It is the first report concerning the ability of curcumin to induce senescence of cells building the vasculature. To our knowledge, there is only one published paper showing that curcumin can induce senescence of primary cells (Hendrayani et al. 2013). Authors of this paper showed that curcumin could induce senescence of cancer-associated fibroblasts (CAF) in a DNA-independent manner, which is consistent with our results. Senescence of these types of cells is recognized as a beneficial effect due to decreased migration/invasion ability. The authors of the paper argued that curcumin-induced senescence could be termed “safe,” because it did not cause DNA DSB, SASP, and protected from metastasis. However, in VSMCs and ECs, at least some protein characteristics for SASP were detected. Previously, we have shown that curcumin can induce senescence of cancer cells, e.g., HCT116. This phenomenon can be considered as important for anti-cancer therapy but the induction of senescence of normal cells would have a negative impact on the organism. However, it has also been postulated that senescence of VSMCs could protect against atherosclerosis (Muñoz-Espín and Serrano 2014). But until now, more evidence supports the contribution of senescence to atherosclerosis progression. It cannot be excluded that the role of senescence depends on the advancement of atherosclerosis and can change with age similar to the role of cellular senescence in general (positive impact in young individuals and detrimental in elderly) (López-Otín et al. 2013). The bioavailability of curcumin is very low, and the activity is short-lived because of its fast metabolism. It was taken for granted that doses not harmful for the organism should not cause time-delayed detrimental effects on the cellular level. However, our results reveal that curcumin concentrations inducing senescence of vascular cells are very close to doses detected in blood after curcumin consumption. We observed cellular senescence after using curcumin in 2.5–5 μM concentrations range, and the highest concentration described in the serum was 3.6 μM (Cheng et al. 2001). Such a concentration was observed after consumption of an extremely high, therapeutic dose, which was administered during cancer therapy. During normal consumption, much lower concentrations of curcumin (nanomolar) are obtained (Anand et al. 2007). Despite the fact that curcumin seems to be a promising agent in slowing-down aging, one should keep in mind that its improved bioavailability and its long-term administration might have an opposite effect and it could accelerate aging, for example by inducing senescence of primary cells building the vasculature.

## Electronic supplementary material

Below is the link to the electronic supplementary material.

Online Resource 1 (Cell cycle analysis of VSMCs and ECs after different doses of curcumin. (**A**) cell cycle of VSMCs (2,5-15 µM curcumin). The representative histograms for 2-day treatment are shown and the graphs for 1 and 2 day-treatment. Accumulation of cells in the G2 phase of the cell cycle was observed upon treatment with curcumin above 7,5 µM. (**B**) cell cycle of ECs (2,5-15 µM curcumin). The representative histograms for 2-day treatment are shown and the graphs for 1 and 2 day-treatment. 1d, 2d – 1, 2 days after curcumin treatment. Error bars indicate SD, n = 3 or more. T test, *- *p*<0.05, **- *p*<0.01, ***- *p*<0.001, compared to control cells. GIF 13.2 mb)

Online Resource 2 The mitotic index of VSMCs and ECs after different doses of curcumin. Percentage of mitotic cells expressed as MPM2-positive cells (**a**) VSMCs (**b**) ECs (1-10 µM curcumin). Summary data and representative dot blots are shown. Noc – nocodazole. (GIF 7.26 mb)

Online Resource 3 DNA methylation in VSMCs (5 µM). The 5-methyl-2′-deoxycytidine (5mdC) content in genomic DNA was expressed as a ratio of 5mdC/(dC+5mdC) [%]. (GIF 2.37 mb)
