# Supplementary figures and images for: Curcumin induces senescence of primary human cells building the vasculature in a DNA damage and ATM-independent manner
Source: Age (Dordr). 2015 Feb 4;37(1):7. doi: 10.1007/s11357-014-9744-y (PMC4315775; doi:10.1007/s11357-014-9744-y)

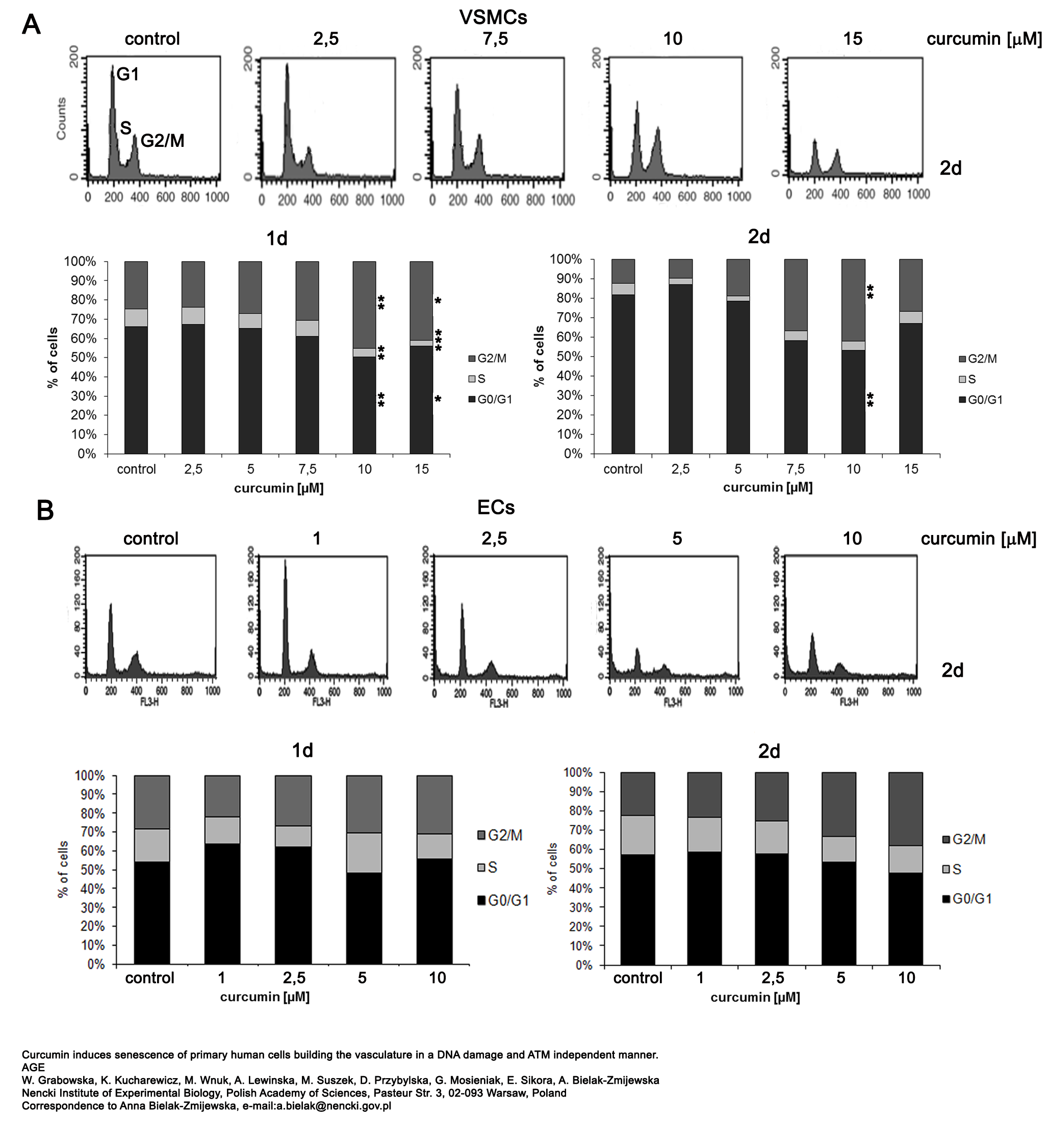

Supplement: Supplementary file 1 — (Cell cycle analysis of VSMCs and ECs after different doses of curcumin. (A) cell cycle of VSMCs (2,5-15 µM curcumin). The representative histograms for 2-day treatment are shown and the graphs for 1 and 2 day-treatment. Accumulation of cells in the G2 phase of the cell cycle was observed upon treatment with curcumin above 7,5 µM. (B) cell cycle of ECs (2,5-15 µM curcumin). The representative histograms for 2-day treatment are shown and the graphs for 1 and 2 day-treatment. 1d, 2d – 1, 2 days after curcumin treatment. Error bars indicate SD, n = 3 or more. T test, *- p<0.05, **- p<0.01, ***- p<0.001, compared to control cells. GIF 13.2 mb) [file 11357_2014_9744_MOESM1_ESM.gif]

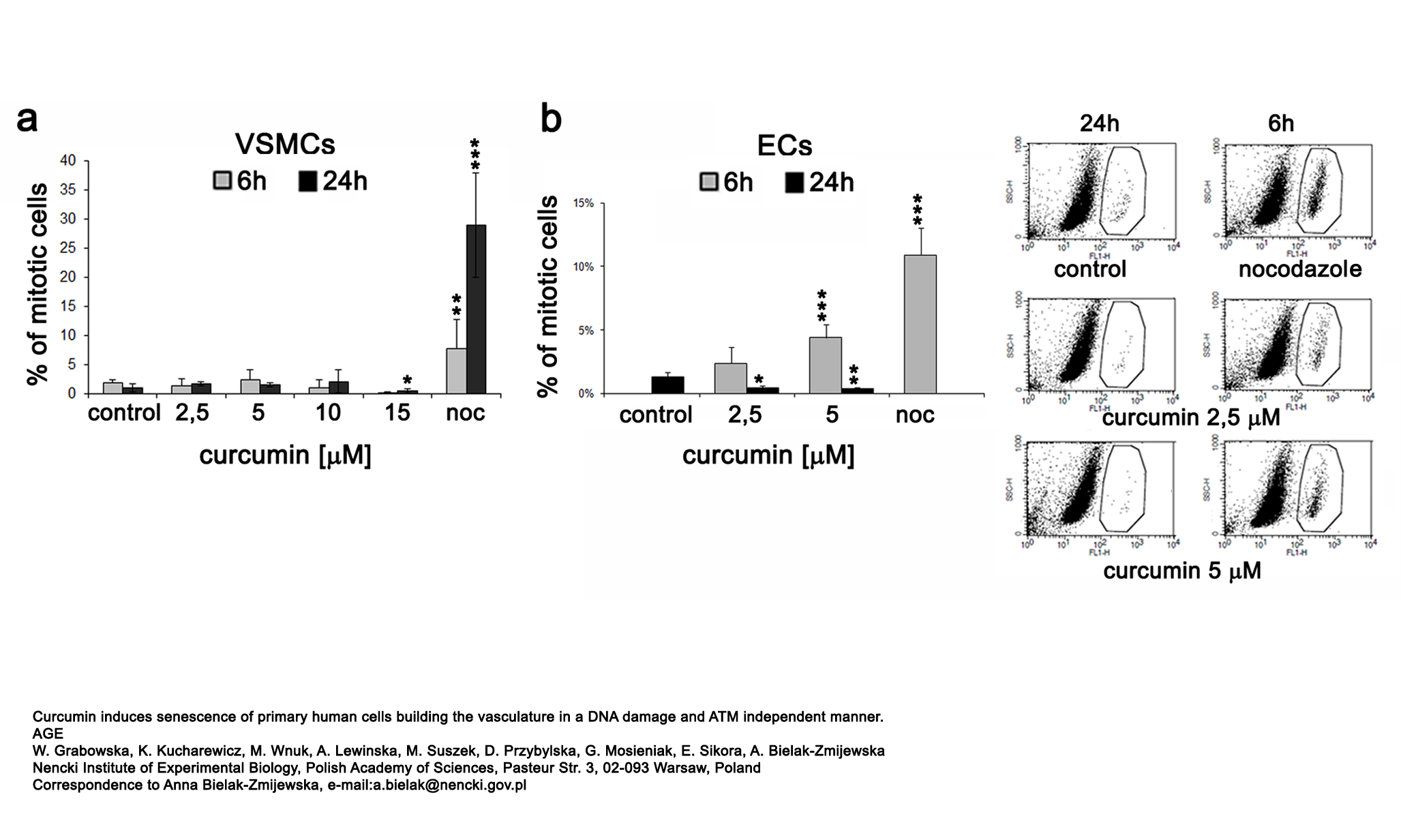

Supplement: Supplementary file 2 — The mitotic index of VSMCs and ECs after different doses of curcumin. Percentage of mitotic cells expressed as MPM2-positive cells (a) VSMCs (b) ECs (1-10 µM curcumin). Summary data and representative dot blots are shown. Noc – nocodazole. (GIF 7.26 mb) [file 11357_2014_9744_MOESM2_ESM.gif]

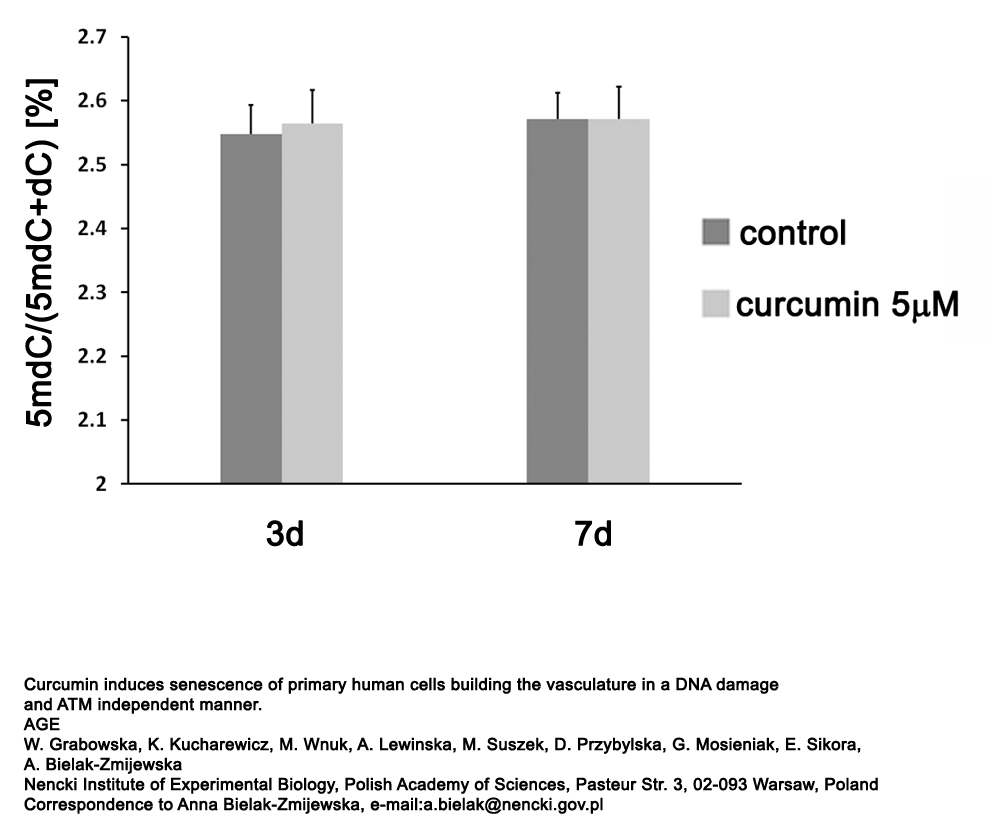

Supplement: Supplementary file 3 — DNA methylation in VSMCs (5 µM). The 5-methyl-2′-deoxycytidine (5mdC) content in genomic DNA was expressed as a ratio of 5mdC/(dC+5mdC) [%]. (GIF 2.37 mb) [file 11357_2014_9744_MOESM3_ESM.gif]
